# A Critical E-box in *Barhl1* 3′ Enhancer Is Essential for Auditory Hair Cell Differentiation

**DOI:** 10.3390/cells8050458

**Published:** 2019-05-15

**Authors:** Kun Hou, Hui Jiang, Md. Rezaul Karim, Chao Zhong, Zhouwen Xu, Lin Liu, Minxin Guan, Jianzhong Shao, Xiao Huang

**Affiliations:** 1 Institute of Cell and Developmental Biology, College of Life Sciences, Zhejiang University, Hangzhou 310058, China; houkun@zju.edu.cn (K.H.); 21507046@zju.edu.cn (H.J.); rezaulkarim@zju.edu.cn (M.R.K.); xenopus@zju.edu.cn (C.Z.); xzwww1994@163.com (Z.X.); 21807042@zju.edu.cn (L.L.); shaojz@zju.edu.cn (J.S.); 2 Department of Biotechnology and Genetic Engineering, Faculty of Biological Sciences, Islamic University, Kushtia 7003, Bangladesh; 3 Institute of Genetics, School of Medicine, Zhejiang University, Hangzhou, Zhejiang 310058, China; gminxin88@zju.edu.cn; 4 Key Laboratory for Cell and Gene Engineering of Zhejiang Province, Hangzhou 310058, China

**Keywords:** *Barhl1*, *Atoh1*, E-box, mESCs, auditory hair cells

## Abstract

*Barhl1*, a mouse homologous gene of Drosophila BarH class homeobox genes, is highly expressed within the inner ear and crucial for the long-term maintenance of auditory hair cells that mediate hearing and balance, yet little is known about the molecular events underlying *Barhl1* regulation and function in hair cells. In this study, through data mining and in vitro report assay, we firstly identified *Barhl1* as a direct target gene of Atoh1 and one E-box (E3) in *Barhl1* 3’ enhancer is crucial for Atoh1-mediated *Barhl1* activation. Then we generated a mouse embryonic stem cell (mESC) line carrying disruptions on this E3 site E-box (CAGCTG) using CRISPR/Cas9 technology and this E3 mutated mESC line is further subjected to an efficient stepwise hair cell differentiation strategy *in vitro*. Disruptions on this E3 site caused dramatic loss of *Barhl1* expression and significantly reduced the number of induced hair cell-like cells, while no affections on the differentiation toward early primitive ectoderm-like cells and otic progenitors. Finally, through RNA-seq profiling and gene ontology (GO) enrichment analysis, we found that this E3 box was indispensable for *Barhl1* expression to maintain hair cell development and normal functions. We also compared the transcriptional profiles of induced cells from CDS mutated and E3 mutated mESCs, respectively, and got very consistent results except the *Barhl1* transcript itself. These observations indicated that Atoh1-mediated *Barhl1* expression could have important roles during auditory hair cell development. In brief, our findings delineate the detail molecular mechanism of *Barhl1* expression regulation in auditory hair cell differentiation.

## 1. Introduction

The auditory sensory epithelium within the mammalian cochlea, the organ of Corti, is elaborately organized with sensory hair cells and non-sensory supporting cells. The hair cells embedded in this sensory organ serve as mechanoreceptor cells for detecting sound and transducing it into electrical signals, and their development and maintenance are critical for auditory function. Any lesion causing degeneration of these sensory cells will lead to hearing loss, which represents the main type of deafness [1]. Nevertheless, the regeneration capacity of mammalian auditory hair cells is extremely limited comparing that of the non-mammalian vertebrates, such as birds and fishes. Spontaneous hair cell regeneration was only reported to occur in the neonatal mice cochlear [2,3]. Therefore, the regeneration of impaired hair cells remains a major challenge in the treatment of deafness by gene and cell therapy.

Atoh1 has been revealed as a master regulator for hair cell development among different phyla. In mammalian, Atoh1 is the earliest known transcription factor expressed in differentiating hair cells and plays essential roles in the differentiation, survival, maturation, and function of hair cells [4,5]. Absence of *Atoh1* will cause apoptosis of progenitor cells, resulting in the incapacity of forming cochlear hair cells [6]. Overexpression of *Atoh1* in embryonic [7,8], neonatal [9,10] and even mature [11,12] mammalian cochlear epithelia can direct the formation of new hair cells, indicating that Atoh1 is sufficient for the induction of hair cells in the cochlea. Furthermore, interestingly enough, supporting cells can be converted into hair cells by upregulating *Atoh1* expression [13], which provides another approach attempted for deafness gene therapy. From the perspective of development, expression pattern of *Atoh1* in the cochlea is in accordance with hair cell differentiation status. *Atoh1* expression can be firstly detected at the base of mouse cochlea between E13.5 and E14.5, and then spreads from the base to the apex of the cochlea. After birth, the expression begins to fade away during the maturation of hair cells and vanishes completely at P6 [14,15]. Surprisingly, sustained expression of *Atoh1* causes hair cells death, leading to hearing loss [16], consistent with what happened when Atoh1 protein degradation was blocked [17]. Several signaling pathways are involved in the regulation of *Atoh1* expression such as Wnt, Notch, and Shh. β-catenin activates *Atoh1* expression through binding to its 3’ enhancer [18], and which can be further facilitated by Wnt pathway [19]. While Notch pathway reduces β-catenin to lower *Atoh1* transcript levels [18]. Moreover, recently described Huwe1 can trigger degradation of Atoh1 [18,20], which plays critical roles both in nerve migration and hair cell specialization [21,22]. In contrast, Shh pathway can prevent Atoh1 degradation by phosphorylating Ser-328 and Ser-339 using PP2A [21]. Taken together, precise spatial and temporal expression of *Atoh1* is extremely critical for hair cell development. Although the functions of Atoh1 are getting much more clear in hair cells, the detailed mechanisms of how it drives their differentiation, survival, and maturation during development still remain largely obscure.

Atoh1 exerts biological effects as a bHLH (basic helix loop helix) transcription factor, which can specifically recognize E-box DNA motif. Previous study has identified the direct targetome of Atoh1 in the cerebellum, one of which is *Barhl1* [23], a member belonging to the BarH class of homeodomain transcription factors. Interestingly, *Barhl1* is also specifically expressed in the inner ear hair cells [24], and functional study has revealed a crucial role for *Barhl1* in the maintenance of auditory hair cells, because targeted deletion of it leads to progressive degeneration of both outer and inner hair cells in the cochlea [25]. Recently, an *in vitro* induction system has been established to evaluate the function of *Barhl1* during hair cell differentiation from mESCs by target disruption on *Barhl1* through Crispr/Cas9 technology [26]. The data clearly showed the inability of *Barhl1* mutated mESCs to differentiate into hair cells in vitro [26], demonstrating the indispensable function of *Barhl1* during the differentiation of hair cells. Thus, it is of great importance to investigate how Atoh1 regulates *Barhl1* to affect hair cell differentiation, which also potentially provides opportunities for manipulating gene expression to drive hair cell fate.

It has been reported that the spatial and temporal expression of *Barhl1* seems to be maintained by both its 5’ and 3’ *Barhl1*-flanking regions and the 5′ promoter region is involved in the auto-regulation of *Barhl1* expression [27]. Atoh1 ChIP-seq data from the cerebellum identify a potential regulatory region in its 3′ flanking region [23]. However, Atoh1 mutated mice have shown loss of *Barhl1* expression in the developing inner ear [15,27,28]. Altogether, Atoh1 is likely to be an important direct upstream activator of *Barhl1* to regulate inner ear hair cell development. Thus, we are proposing that the molecular mechanism governing *Barhl1* expression in cochlea hair cells is mostly depended on binding of Atoh1 on the 3′ *Barhl1*-flanking region. To verify our speculation, we first compared the previously reported cerebellar Atoh1 targetome [23] with RNA-seq data from the developing inner ear [29] in detail. We identified an essential region on *Barhl1* 3′ enhancer, which contains multiple E-boxes potential for Atoh1 binding. We further distinguished one crucial E-box by mutational assays in *in vitro* cultured HEK293T cells. Secondly, we created disruptions on *Barhl1* locus targeting the essential E-box by CRISPR/Cas9 technology in mESCs and established the *Barhl1* E3-box mutated (BEM) mESC line. Thirdly, the BEM mESCs were further induced to inner ear hair cell fate following our efficient stepwise in vitro differentiation system to explore the pivotal roles of this E-box in hair cell development. Finally, although the mutation of *Barhl1* causes deafness in vivo [25] and incapacity to differentiate into hair cell-like cells in vitro [26], the molecular mechanisms of *Barhl1* in hair cell fate and function still keep largely unknown. We; thus, defined the accurate start point of *Barhl1* expression in the induction system and performed RNA-seq analysis of induced hair cells from BEM mESCs at the critical time. Further analysis of expression profiles and bioinformatics predictions of the direct targets revealed the potential functions and mechanisms of *Barhl1* in hair cell functions and development. Taken together, here we reveal that Atoh1 regulates the expression of *Barhl1* through binding to a specific E3 box, which in turn affects hair cell development. Furthermore, our data may provide critical insights into gene or stem cell therapies for *Barhl1*-related hereditary deafness.

## 2. Materials and Methods

### 2.1. Plasmid Construction and Site-Directed Mutagenesis

Atoh1 expression plasmid: The pCS107-Atoh1 expression plasmid included 1056 bp mouse Atoh1 coding region inserted downstream of CMV IE94 promoter. *Barhl1* reporter plasmid: The *Barhl1* enhancer-n green fluorescent protein (nGFP) plasmid was constructed using the 318 bp Atoh1-bound region located 800 bp away from 3’ of the stop codon of *Barhl1* to replace the Atoh1 enhancer in Atoh1 enhancer-nGFP reporter (J. Johnson Lab, Dallas, TX, USA). Point mutations in the *Barhl1* reporter plasmid were introduced using the Finnzyme Phusion site-directed mutagenesis kit (Thermo Fisher Scientific, Wilmington, DE, USA).

### 2.2. Reporter Assay

HEK293T cells were cultured in Dulbecco’s modified eagle’s medium (DMEM, Gibco, Carlsbad, CA, USA) supplemented with 10% fetal bovine serum (FBS, Gibco, Carlsbad, CA, USA). Reporter assays were conducted using HEK293T cells (2 × 10^5^) seeded in each well of a 24-well plate and transfected 24 h later with 170 ng *Barhl1* reporter plasmid (wild type or E-box mutation constructs) and 330 ng pCS107-Atoh1 plasmid, or empty plasmid control using Lipofectamine 2000 (Invitrogen, Carlsbad, CA, USA) according to the manufacturer’s instructions. In addition, 500 ng CMV–red fluorescent protein (RFP) plasmid was also transfected into a control well to assess the transfection efficiency. At 48 h post transfection, fluorescent imaging was performed with a Zeiss Axio Observer A1 (Zeiss, Oberkochen, Germany).

### 2.3. mESC Cell Culture and Differentiation

The maintenance and differentiation of mESCs were conducted in accordance with a step-by-step protocol performed previously [26]. In brief, firstly the early primitive ectoderm-like (EPL) cells were induced from mESCs for 7 days in Dulbecco’s modified eagle’s medium: Nutrient mixture F-12 (DMEM/F12, Gibco, Carlsbad, CA, USA) supplemented with 10% FBS (Gibco, Carlsbad, CA, USA), 0.1 mM non-essential amino acids (Invitrogen, Carlsbad, CA, USA), 55 mM 2-mercaptoethanol (Invitrogen, Carlsbad, CA, USA), 2 mM L-glutamine (Invitrogen, Carlsbad, CA, USA), and 50% MEDII. MEDII is a conditioned medium which was collected after 4 days of culture of Hep G2 cells in DMEM supplemented with 10% FBS and 1mM GlutaMAX™ supplement. Secondly, the EPL cells were transferred into DMEM/F12 (Gibco, Carlsbad, CA, USA) supplemented with N2 and B27 (Invitrogen, Carlsbad, CA, USA), fibroblast growth factor 3 (FGF3, R&D Systems, Minneapolis, MN, USA, 50 ng/mL), fibroblast growth factor 10 (FGF10, R&D Systems, Minneapolis, MN, USA, 50 ng/mL), epidermal growth factor (EGF, Gibco, Carlsbad, CA, USA, 25 ng/mL), insulin-like growth factor-1 (IGF-I, Gibco, Carlsbad, CA, USA, 10 ng/mL), and heparan sulfate (Sigma, St.Louis, MO, USA, 50 ng/mL) for 14 days to generate otic progenitors. Lastly, the otic progenitors were dissociated and seeded on inactivated embryonic chicken utricle stromal cells. Co-cultures were grown for 30 days using DMEM/F12 (Gibco, Carlsbad, CA, USA) supplemented with N2 and B27 (Invitrogen, Carlsbad, CA, USA), EGF (Gibco, Carlsbad, CA, USA, 25 ng/mL) and retinoic acid (RA, Sigma, St.Louis, MO, USA, 1 µM).

### 2.4. Generation of BEM mESC Lines

The targeted disruption on the E3 site among *Barhl1* 3′-UTR region in mESCs was performed using CRISPR/Cas9 technology. Target guide sequences were designed using the CRISPR design tool (http://www.genome-engineering.org). The sequences of oligos are as follows: 5′-CACCGCAGGGCCGGGCACCAGCTGC-3′ and 5′-AAACGCAGCTGGTGCCCGGCCCTGC-3′. Oligos were annealing and cloned into LentiCRISPRv2 (Addgene, Beijing, China) to produce CRISPR/Cas9 vector specifically targeting the E3 site. To produce the lentivirus, 1.11 µg respective CRISPR/Cas9 construct was co-transfected into HEK293T cells with 0.56 µg pMD2.G and 0.83 µg psPAX2 vectors. The lentivirus was then used to infect mESCs, and cells were selected using 0.8 µg/mL puromycin. Puromycin-resistant colonies were picked and expanded. Genotyping was performed by PCR with primer pairs 5′-GCAGCCAGACCTCTTGGGTTATC-3′ and 5′-AGGATGGAGAGAGAGCAAAGGGT-3′. The PCR products were TA cloned and sequenced. Several most likely off-target sites predicted using the CRISPR design tool were also selected for off-target examination at the same time. The detail information of selected off-target sites were listed in Table S1, and the primers used for off-target examinations were listed in Table S2. All PCR products were sent to BioSune (Biotech, Shanghai, China) for sequencing.

### 2.5. RNA Extraction and Real-Time Quantitative PCR (qPCR)

Total RNA was isolated from cells using TRIzol reagent (Life Technologies, Carlsbad, CA, USA) and reverse transcription of 0.5 to 2 μg total RNA was performed using the Moloney Murine Leukemia Virus (M-MLV) Reverse Transcriptase Kit (Fermentas, Waltham, MA, USA). Quantitative PCR analysis was performed in triplicate with Synergy Brands (SYBR) Green Supermix (Bio-Rad) on a Computational Fluid Dynamics X (CFX) Connect Real-Time PCR System (Bio–Rad, Hercules, CA, USA). Relative gene expression level was calculated by using the comparative ΔΔCt method [30] and was normalized with glyceraldehyde-3-phosphate dehydrogenase(GAPDH). The primers flanking an intron used in qPCR are listed in Table S3.

### 2.6. Western Blot

Protein extracts were prepared using Radio-Immunoprecipitation Assay (RIPA) buffer (Beyotime, Shanghai, China) supplemented with 1 mM phenylmethanesulfonyl fluoride (PMSF). Proteins were separated in 12% polyacrylamide gels and transferred to polyvinylidene fluoride (PVDF) membranes (Millipore, Etobicoke, Ontario M9W 6Y1, Canada). Membrane was blocked with 5% bovine serum albumin (BSA) in Tris buffered saline with 0.1% Tween-20 (TBST) and incubated overnight at 4 °C with primary antibodies diluted in TBST: Barhl1 (1:500, mouse polyclonal, Abcam, Shanghai, China) and Glyceraldehyde-3-phosphate dehydrogenase (GAPDH) (1:500, mouse monoclonal, Goodhere, Hangzhou, China). Membranes were washed with TBST and incubated with horseradish peroxidase-conjugated anti-mouse secondary antibody (1:1000, Beyotime, Shanghai, China) for 1 h at room temperature. Immunopositive bands were detected using the enhanced chemiluminescent reagent (Thermo Fisher Scientific, Wilmington, DE, USA).

### 2.7. Alkaline Phosphatase (AP) and Immunofluorescence Staining

Alkaline Phosphatase Staining Kit (Stemgent, Shanghai, China) was used for assessing AP activity of mESCs. For immunofluorescence (IF) staining, cells were fixed with 4% paraformaldehyde in phosphate buffer saline (PBS) for 15 min at room temperature, and then were permeabilized with 0.25% TritonX-100 in PBS for 10 min. Fixed and permeabilized cells were incubated and blocked by 1% BSA in PBS containing 0.1% Tween-20 for 1 h. Primary antibodies incubation were performed overnight at 4 °C. Specific primary antibodies were diluted in 1% BSA in PBS containing 0.1% Tween-20. The primary antibodies included Oct4 (1:100, rabbit, Abcam, Shanghai, China), Nanog (1:500, rabbit, Abcam, Shanghai, China), Sox2 (1:500, rabbit, Abcam, Shanghai, China), Brn3c (1:50, mouse, Santa Cruz Biotechnology, Santa Cruz, USA), Pax2 (1:200, rabbit, Abcam, Shanghai, China), Pax8 (1:50, mouse, Abcam, Shanghai, China), Espin (1:50, rabbit, Santa Cruz Biotechnology, Santa Cruz, USA), and Myo7a (1:200, rabbit, Abcam, Shanghai, China). After washing three times in PBS for 15 min each, Alexa Fluor 594- or Alexa Fluor 488- conjugated secondary antibodies (1:500, Jackson ImmunoResearch, Shanghai, China) were used to detect the signals with 4′,6-diamidino-2-phenylindole (DAPI) to show the nuclei. For visualization of the cytoskeleton structure, Phalloidin-iFluor™ 594 Conjugate (1:1000, AAT Bioquest, Hangzhou, China) was incubated for 1 h to detect F-actin in cells. After incubating and washing, the cells were imaged on a Zeiss LSM 710 (Zeiss, Oberkochen, Germany) scanning confocal microscope. Quantification of positive-stained cells was based on a proportion of total number of induced cells by counting approximately 500 cells in 10 randomly selected regions and repeated in biological triplicate.

### 2.8. FM1-43FX Uptake Studies

The cells were stained with 5 µM fluorescent dye FM1-43FX (Invitrogen, Carlsbad, CA, USA) which is a reliable reporter to evaluate the hair cell viability after being washed twice with Hanks’ Balanced salt solution (HBSS) at room temperature. Following this, the cells were rinsed in HBSS solution three times. Then the samples were fixed with 4% paraformaldehyde solution and also stained with Myo7a. DAPI was used to indicate the nuclei. All fields were imaged with a Zeiss LSM 710 scanning confocal microscope. Quantification of positive-stained cells was performed as previously described.

### 2.9. RNA-Sequencing (RNA-Seq) and Data Analysis

Total RNA from cells was isolated using TRIzol reagent (Life Technologies, Carlsbad, CA, USA) according to a standard protocol and was quantified with a Nanodrop spectrophotometer (Thermo Fisher Scientific, Wilmington, DE, USA). After preparing RNA-seq library with the Illumina TruseqTM RNA Sample Prep Kit, the samples were sequenced on the Illumina HiSeq 4000 150 bp Paired-End Platform in MajorBio (Shanghai, China). For analysis, the reads generated were mapped to the mouse genome (GRCm38.84) using TopHat2 with default setting. EdgeR was used to standardize the gene read counts and a negative binomial test was used as a basis to determine genes with different expressions. Genes with significantly different expressions (FDR < 0.05 and |log2FC| ≥ 1) were selected to be further processed. Additionaly, gene ontology (GO) enrichment analysis of differentially expressed genes was finished by the DAVID functional annotation tool (http://david.abcc.ncifcrf.gov/).

### 2.10. Statistical Analysis

At least three biological replicates were analyzed for the statistics presented. Results are expressed as means ± standard error of the mean (SEM). An unpaired Student’s t-test was used to assess the statistical significance which was set at *p* values < 0.05.

## 3. Results

### 3.1. Barhl1 Is a Direct Downstream Target of Atoh1 in the Inner Ear Hair Cells

**Figure 1:**
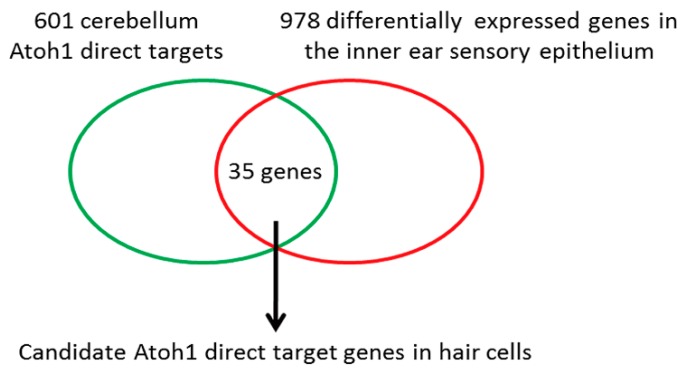
Potential Atoh1 direct target genes in hair cells. Schematic shows that by cross-referencing the 601 cerebellum Atoh1 direct target genes previously identified by Klisch et al. (2011) and 978 differentially expressed genes in the inner ear sensory epithelium identified by Scheffer et al. (2015), 35 genes were selected as candidates Atoh1 direct targets in hair cells.

*Atoh1* is an essential master gene for hair cell development. However, the molecular mechanism of how Atoh1 drives hair cell differentiation remains unclear since little is known about Atoh1 direct targets in hair cells. To identify potential direct targets of Atoh1 in the auditory hair cells, we compared the mouse cerebellar Atoh1 targetome [23] with RNA-seq data from the inner ear hair cells during development [29]. We overlapped the top 601 Atoh1 direct targets from the cerebellar granule neuron precursors (*p* ≤ 0.01) and the 978 genes that are differentially expressed in developing hair cells (*p* ≤ 0.05). We found that 35 of 601 Atoh1 direct targets in the developing cerebellum also showed cell type specific expression in the inner ear sensory epithelium, of which 23 were significantly upregulated, while 12 were significantly downregulated in hair cells (Figure 1, Table 1). Among these candidate genes, *Srrm4* [31] and *Atoh1* itself [32] have been known to be direct target genes of Atoh1 in hair cells. Furthermore, the resultant 35 identified candidate Atoh1 direct targets also included genes that had been previously reported to play essential roles in hair cells, such as *Barhl1* [25], *Srrm4* [33], and *Atoh1* itself. Interestingly, most of the rest genes remained unclear as to whether there were any associations with hair cells functionally.

The BarH class homeobox gene *Barhl1* among the 35 predicted Atoh1 direct targets in hair cells is of our special interest (Table 1). As shown in the cerebellar Atoh1 targetome and inner ear RNA-seq datasets, *Barhl1* is one target of Atoh1 in the developing cerebellum (*p* = 8.91 × 10^−5^), and in auditory sensory epithelia its transcript has nearly 190-fold enrichment in Atoh1-expressing hair cells (*p* = 9.13 × 10^−9^) [23,29]. In addition, *Barhl1* has been previously known to be critical for the maintenance of hair cells because the absence of *Barhl1* leads to degeneration of auditory hair cells and thus hearing loss [25]. These data strongly suggest that *Barhl1* is a direct downstream target of Atoh1 in hair cells.

### 3.2. Atoh1 Binds to an Essential E-box in Barhl1 3’-Flanking Enhancer

**Figure 2:**
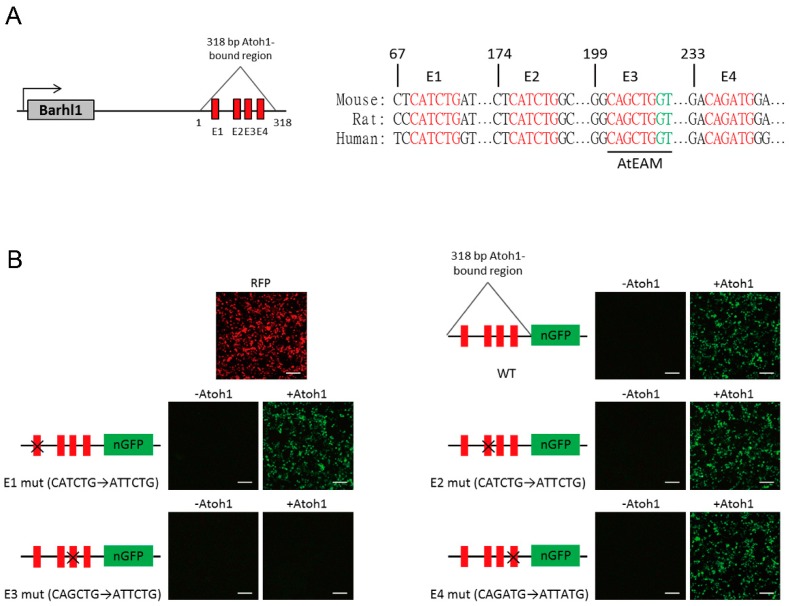
Atoh1 activates *Barhl1* expression through an essential E-box motif in the 3′ enhancer region. (**A**) Schematic of the 318 bp Atoh1-bound region downstream of *Barhl1* indicating the E-boxes and their conservation among mouse, rat, and human. (**B**) Transcriptional activity of Atoh1 on E-box mutation constructs of the *Barhl1* 3’ enhancer. Reporter contains the *Barhl1* enhancer (Wild Type, WT) or the enhancer carrying an E-box mutation (E1 mut, CATCTG was mutated to ATTCTG; E2 mut, CATCTG was mutated to ATTCTG; E3 mut, CAGCTG was mutated to ATTCTG and E4 mut, CAGATG was mutated to ATTATG) was co-transfected with a control (empty vector) or a plasmid expressing Atoh1. Expression of red fluorescent protein (RFP) indicated the efficiency of transfection. The enhancer-reporter activity was determined by n green fluorescent protein (nGFP) fluorescence after 48 h. Results indicate that this E3 site is critical for Atoh1-mediated *Barhl1* activation. Scale bars, 200 µm.

To identify candidate enhancer region of *Barhl1*, we explored the Atoh1-bound region on the *Barhl1* locus from mouse cerebellum Atoh1 ChIP-seq data [23]. An Atoh1-bound 318 bp genomic sequence within *Barhl1* 3′–flanking region was found, which is located about 0.8 kb to the translation stop codon for *Barhl1*. This region contains four highly conserved Atoh1 putative binding sites (E-boxes) named from E1 to E4 (Figure 2A). This indicates that Atoh1 might directly regulate *Barhl1* expression through this potential enhancer.

To test whether this candidate *Barhl1* enhancer is responsive to Atoh1, we placed it in front of the nGFP reporter plasmid which was then co-transfected with either empty vector or Atoh1 expression vector into HEK293T cells. As expected, the candidate *Barhl1* enhancer-reporter was activated by the co-transfection with Atoh1 expression vector (Figure 2B), confirming that it has Atoh1-mediated enhancer activity.

In addition, to further determine the Atoh1 binding motifs in this identified *Barhl1* enhancer region, we created mutations on the E1–E4 sites of the *Barhl1* enhancer-reporter construct respectively. Co-transfection assays in HEK293T cells demonstrated that in the presence of Atoh1 expression, mutations of E1, E2, and E4 sites had no effect on the expression of nGFP, while mutation of the E3 site completely abolished the Atoh1-mediated enhancer-reporter activity (Figure 2B). This indicates that the E3 site is critical for stimulative activity of Atoh1 on the *Barhl1* enhancer. Notably, the E3 E-box with its flanking sequence (CAGCTGGT) is consistent with the previously identified consensus-binding motif for Atoh1 (AtEAM) [23], suggesting that Atoh1 can bind to this essential motif to activate *Barhl1* expression directly.

### 3.3. Establishment of BEM mESC Line Carrying the E3 Motif Mutant

**Figure 3:**
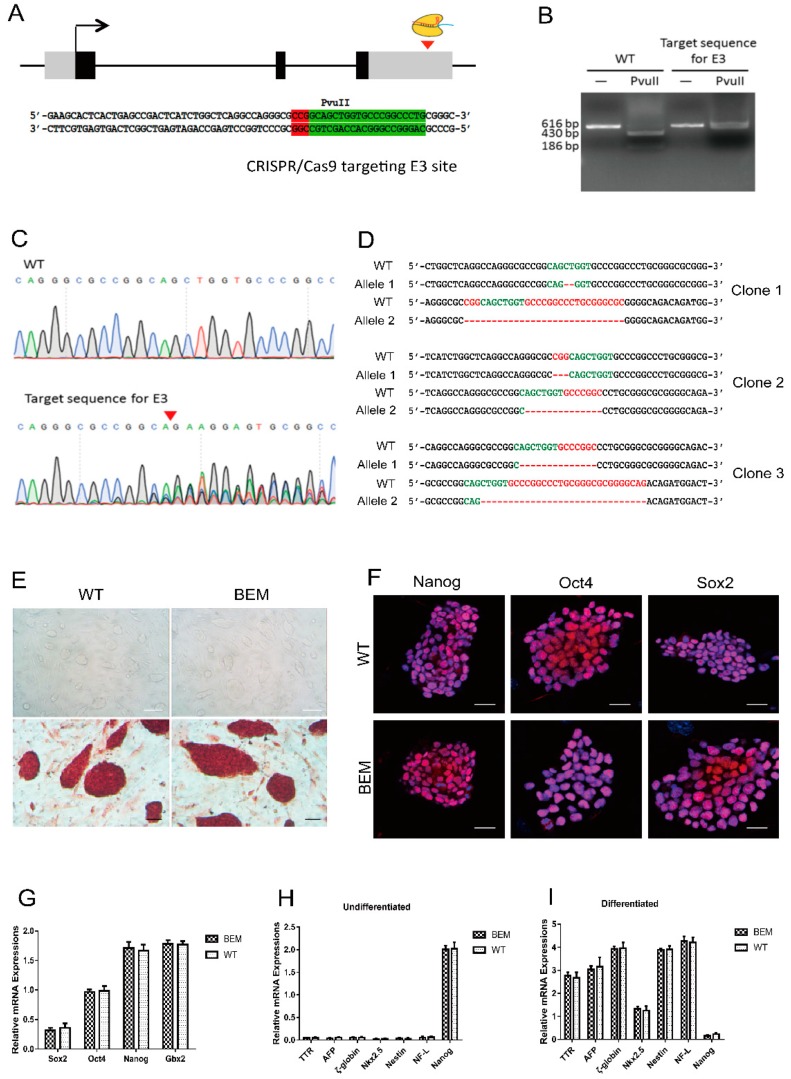
Generation and characterization of *Barhl1* E3-box mutated (BEM) mouse embryonic stem cell (mESC) lines. (**A**) Schematic diagram showing the position of target site of CRISPR/Cas9 in the *Barhl1* E3 site. Gray/black boxes, exons; black boxes, *Barhl1* coding sequence (CDS); solid line between black boxes, introns; black arrow, direction of transcription; red arrowheads, indicating target sites of CRISPR/Cas9 in the *Barhl1* E3 site. Sequence of target sequence for the E3 site (indicated by green) carrying a cleavage site of PvuII and the protospacer adjacent motif (PAM, indicated by red) were displayed in detail. (**B**) Restriction digestion of 616bp polymerase chain reaction (PCR) product of the E3 site by PvuII. The WT E3 site instead of the mutated E3 site can be digested into 430 and 186 bp. PCR products without enzymatic digestion were used as controls. (**C**) The sequencing peak map of the WT E3 site and the mutated E3 site. Multiple peaks were shown at the mutated E3 site instead of a single peak in the WT E3 site. (**D**) Sanger sequencing revealed compound heterozygous E3 site mutations occurred in all three selected clones, Clone1 (29 and 2 bp deletion), Clone2 (14 and 4 bp deletion) and Clone3 (30 and 14bp). Clone1 was picked for further analysis and named as BEM line. (**E**) Phase contrast microscopy and alkaline phosphatase (AP) staining of WT mESCs and BEM line. Scale bars, 25 µm. (**F**) Immunostaining analyses of embryonic stem cell (ESC) markers Nanog, Oct4 and Sox2 in WT mESCs and BEM line. Scale bars, 20 µm. (**G**) Quantitative Real-Time polymerase chain reaction (qRT-PCR) analyses for expression of ESC markers Sox2, Oct4, Nanog, and Gbx2 in WT mESCs and BEM line. No significant differences in the mRNA expression levels of *Sox2*, *Oct4*, *Nanog,* and *Gbx2* between both kinds of mESCs were found. Data are mean ± SEM (*n* = 3). (**H**,**I**) qRT-PCR analyses for expression of germ layer-specific markers *AFP* and *TTR* (endoderm), *ζ-globin* and *Nkx2.5* (mesoderm), *Nestin* and *NF-L* (ectoderm), and ESC marker *Nanog*. RNA was isolated from the undifferentiated WT mESCs and BEM line (**H**), and cells after spontaneous differentiation (**I**). All of three germ layer-specific markers were found to be upregulated upon spontaneous differentiation of two kinds of mESCs, and no significant differences in their transcript levels were found. Undifferentiated: The undifferentiated mESCs; differentiated: The in vitro spontaneously differentiated cells. Data are mean ± SEM (*n* = 3).

We recently reported that *Barhl1* is indispensable for hair cell differentiation in the *in vitro* induction system from mESCs, where the mutation of *Barhl1* was created on the CDS region by Crispr/Cas9 technology [26]. Thus, to evaluate the importance of this E3 motif for hair cell development, it is of great interest to compare the effects of the CDS region and the E3 motif mutations on hair cell differentiation in our *in vitro* induction system. We attempted to create mutation in mESCs on the E3 site which can be restriction digested by PvuII in *Barhl1* enhancer by Crispr/Cas9 technology (Figure 3A). Lentiviral CRISPR/Cas9 vectors were constructed to specifically target the E3 site in *Barhl1* enhancer. After lentivirus transfection and puromycin selection, survival colonies were picked and expanded for genotyping. The 616 bp PCR product of the wild type (WT) E3 site while not the mutated E3 site can be digested into 430 and 186 bp due to the unrecognition of the mutated E3 site by PvuII (Figure 3B). The multiple peaks in the sequencing peak map also indicated successful mutation creations on the E3 site (Figure 3C). Compound heterozygous mutations were introduced in all the clones sent for sequencing (Figure 3D). Among them, clone 1 was designated as BEM since it carries frameshift mutations in both alleles (2 and 29 bp deletion, respectively). At the same time, the possibility of off-target editing was also evaluated by genome typing from the top five most probable off-target sites. Additionally, the data showed that no mutations were found among these loci in the BEM line (Table S1; Figure S1).

### 3.4. Disruption of the Barhl1 E3 Site Does Not Affect the Pluripotency of mESCs

To avoid the potential effects of the E3 motif mutation on mESCs, we conducted several pluripotency validations of the BEM mESC line. When the BEM line were cultured on mouse embryonic fibroblast (MEF) feeder in mESC maintenance medium, typical WT ES-like colonies with strong alkaline phosphatase activity could be formed (Figure 3E). Additionally, quantitative real-time polymerase chain reaction (qRT-PCR) analysis confirmed that there were no significant differences between WT mESCs and BEM line on the expression levels of ESC markers, including *Sox2*, *Oct4*, *Nanog,* and *Gbx2* (Figure 3G). The BEM cells were also confirmed to be positive for the pluripotency marker genes such as *Oct4*, *Sox,2* and *Nanog* by immunofluorescence at the same time (Figure 3F).

To further examine their pluripotency, the BEM line cells were cultured in suspension to test the capacity to form embryoid bodies (EBs) and differentiate to EB-derived cells. Our data showed there were no obvious differences on the capacity to form EBs between WT mESCs and BEM line, and the expression of three different germ layers specific markers (*AFP*, *TTR* for endodermal markers; *ζ-globin*, *Nkx2.5* for mesodermal markers, and *Nestin*, *NF-L* for ectodermal markers) were upregulated obviously in both cell lines (Figure 3H,I). And at the transcription level, these markers also displayed no significant differences between these two cell lines (Figure 3H,I). Taken together, our data indicate that the BEM line has the potential of multi-lineage differentiation *in vitro* just like WT mESCs. Thus, disruption of the *Barhl1* E3 site in mESCs does not affect its stem cell characteristics.

### 3.5. Disruption of the Barhl1 E3 Site Does Not Affect EPL Cell Induction and Otic Progenitor Differentiation

**Figure 4:**
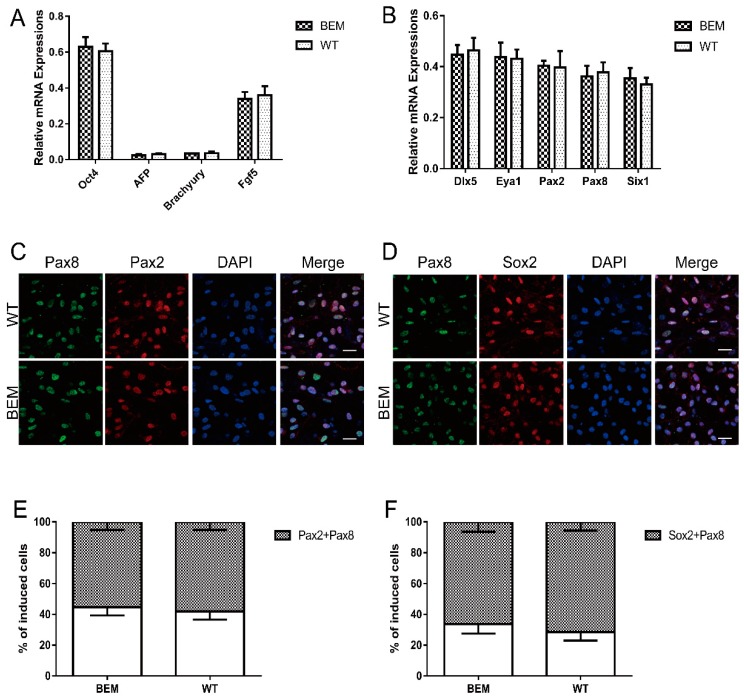
Targeted disruption of the essential E-box in *Barhl1* enhancer does not affect induction of EPL cells and otic progenitors from mESCs. (**A**) qRT-PCR analyses for expression of pluripotent cell-related marker *Oct4*, visceral endoderm-related marker *AFP*, mesoderm-related marker *Brachyury,* and primitive ectoderm-related marker *Fgf5* in cell aggregates derived from WT and BEM mESCs on day 7. There were no significant differences in the mRNA expression levels of *Oct4* and *Fgf5* between two kinds of mESC-derived cells and no significant expressions of *AFP* and *Brachyury* in these induced cells, indicating the transition of both mESCs to EPL cells. Data are mean ± SEM (*n* = 3). (**B**) qRT-PCR analyses for expression of otic markers *Dlx5*, *Eya1*, *Pax2*, *Pax8,* and *Six1* in WT-and BEM-derived cultures at day 21. No significant differences in the mRNA expression levels of *Dlx5*, *Eya1*, *Pax2*, *Pax8,* and *Six1* between both kinds of mESC-derived cells were detected. Data are mean ± SEM (*n* = 3). (**C**,**D**) Immunostaining analyses of otic markers Pax2/Pax8 (**C**) and Pax8/Sox2 (**D**) in WT- and BEM-derived cultures at day 21. Scale bars, 20 μm. (**E**,**F**) The percentages of double-immunopositive cells for Pax2/Pax8 (E) and Pax8/Sox2 (F) in WT- and BEM-derived cultures at day 21. There were no significant differences in the percentages of double-immunopositive cells for Pax2/Pax8 and Pax8/Sox2 between these two mESC-derived cells. Data are mean ± SEM (*n* = 3).

Following the stepwise induction, for the same reason, we subsequently examined the efficiency of BEM line differentiation into EPL cells and otic progenitors. We exposed the BEM line and WT mESCs (which were operated as controls) to the *in vitro* hair cell induction system to generate auditory hair cell-like cells. After seven days of induction, we examined the expression levels of several marker genes by qRT-PCR analyses. In WT mESCs and BEM line derived cells, high levels of *Oct4* (pluripotency marker) and *Fgf5* (primary ectodermal marker) were detected, while *AFP* (endodermal marker) and *Brachyury* (mesodermal marker) had much lower levels of transcription, indicating that both lines of mESCs have converted into EPL cells (Figure 4A). In addition, given no significant differences in transcript levels of all of the above marker genes between WT mESCs and BEM mESCs derived cells (Figure 4A), the conversion of each mESC line to EPL cells is comparable and disruption of the E3 site has no effect on the induction of EPL cells from mESCs. After EPL cell induction, the cell aggregates were transferred into medium containing FGF3, FGF10, EGF, and IGF-I and cultured until day 21. To define whether the induced cells possess committed otic progenitor fates, inner ear progenitor markers (including *Dlx5*, *Eya1*, *Pax2*, *Pax8*, *Sox2,* and *Six1*) [34,35] were quantitatively and qualitatively analyzed by qRT-PCR and fluorescent immunostaining. After 14 days of induction, abundant expressions of *Dlx5*, *Eya1*, *Pax2*, *Pax8,* and *Six1* were detected but did not differ significantly between WT- and BEM-derived cells (Figure 4B). Moreover, immunofluorescence double staining was performed to detect the co-expression of otic progenitor-related markers Pax2, Pax8, and Sox2. Additionally, results showed that a large number of Pax2 and Pax8 double positive cells were found both in WT- and BEM-derived populations, reaching 58.1 ± 5.2% and 55.3 ± 5.3%, respectively (Figure 4C, E). The ratios of Pax8 and Sox2 double positive cells in WT- and BEM-derived populations were even much higher and reached 71.4 ± 5.6% and 66.2 ± 6.3%, respectively (Figure 4D,F). Statistically, the above data revealed no significant differences in the proportion of double staining cells between WT- and BEM-derived populations (Figure 4E,F). Taken together, our results clearly suggest that disruption of the essential E-box has no impact on the inductions toward EPL cells and otic progenitors from mESCs.

### 3.6. Disruption of the Barhl1 E3 Site Affects Auditory Hair Cell Differentiation

**Figure 5:**
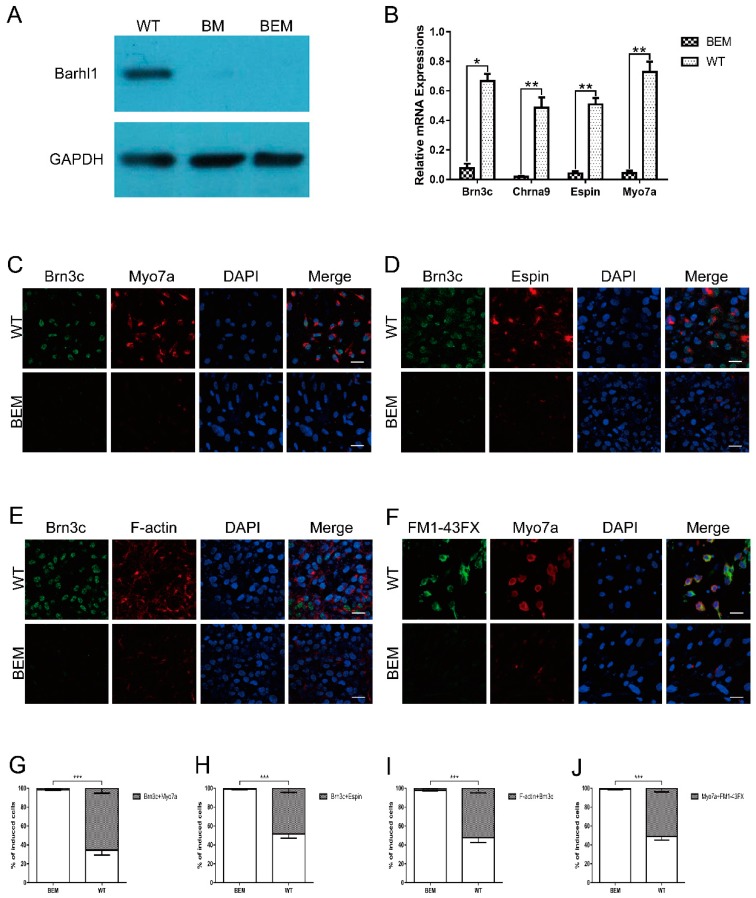
Targeted disruption of the essential E-box in *Barhl1* enhancer affects *Barhl1* expression and the differentiation of hair cell-like cells. (**A**) Western blot analysis of *Barhl1* expression in WT- and BEM-derived cultures at day 51. BM-derived cultures were used as a negative control. Barhl1 protein was absent in BM derivatives and BEM derivatives. (**B**) qRT-PCR analyses for expression of hair cell-specific markers *Brn3c*, *Chrna9*, *Espin,* and *Myo7a* in WT- and BEM-derived cultures at day 51. There was a significant reduction in mRNA expression levels of *Brn3c*, *Chrna9*, *Espin,* and *Myo7a* in BEM-derived cells. Data are mean ± SEM (*n* = 3). *p < 0.05. ** *p* < 0.01 (**C**–**F**) Immunostaining for hair cell-specific markers Brn3c/Myo7a (**C**) and Brn3c/Espin (**D**), phalloidin staining combined with immunostaining for Brn3c (**E**), and FM1-43FX staining combined with immunostaining for Myo7a (**F**) in WT- and BEM-derived cultures at day 51. Scale bars, 20 µm. (**G**–**J**) The percentages of double-positive cells for Brn3c/Myo7a (**G**), Brn3c/Espin (**H**), Brn3c/F-actin (**I**), and FM1-43FX/Myo7a (**J**) in WT- and BEM-derived cultures at day 51. Significant reductions in the percentages of double-positive cells for Brn3c/Myo7a, Brn3c/Espin, Brn3c/F-actin, and FM1-43FX/Myo7a in BEM-derived cells were found. Data are mean ± SEM (*n* = 3). ***p < 0.001.

The *in vitro* induction system for auditory hair cells involves several sequential steps which mimic the *in vivo* developmental process. After ruling out the possibilities of the early steps, the impacts on the last step were certainly evaluated to confirm the indispensable function of this E3 motif. WT- and BEM-derived otic progenitors obtained above were co-cultured respectively on the embryonic chicken utricle stromal cells in medium supplemented with EGF and RA. After 30 days of co-culture, the protein levels of *Barhl1* in WT- and BEM-derived cell populations were determined by western blot. The BM cell line [26] carrying a targeted disruption on the *Barhl1* CDS served as a control. As anticipated, Barhl1 protein can only be detected in WT derivatives and was absent in BM and BEM derivatives (Figure 5A), suggesting that this E3 motif is definitely required for *Barhl1* expression. Simultaneously, as Barhl1 has been reported to be essential for the maintenance of hair cells [25], to characterize the derived cell lineage identity, the transcript abundances of hair cell-specific markers such as *Brn3c*, *Chrna9*, *Espin,* and *Myo7a* [34,36,37] were examined in both WT- and BEM-derived cell populations. Strikingly, qRT-PCR results revealed large-scale declines in the transcription of *Brn3c*, *Chrna9*, *Espin,* and *Myo7a* compared with WT control (Figure 5B). The induction efficiency was determined by counting the proportion of induced hair cell-like cells in the population. Co-expressions of Brn3c, Myo7a, and Espin in the cell populations were displayed by immunofluorescence. The double staining data demonstrated the proportion of Brn3c and Myo7a double positive cells in BEM derivatives dramatically decreased from 65.5 ± 5.2% to 1.8% ± 0.5% (Figure 5C,G). Similarly, the percentage of induced cells co-expressing Brn3c and Espin in BEM derivatives also declined apparently from 48.3 ± 4.5% to 0.9 ± 0.4% relative to WT derivatives (Figure 5D,H). Next, WT- and BEM-derived cells were also stained with phalloidin to check the distinctive stereocilia-like structures. The ratio of cells expressing both F-actin and Brn3c in WT derivatives was around 52.5 ± 4.9%, while in BEM derivatives the ratio was dramatically reduced to 2.1 ± 0.5% (Figure 5E,I). Consistent with results above, FM1-43FX uptake studies demonstrated that the proportion of Myo7a-positive cells absorbing FM1-43FX meanwhile reached up to 51.1 ± 3.9% in WT derivatives. However, in BEM derivatives, there was little detectable presence of cells absorbing FM1-43FX, which was also positive for Myo7a with a ratio of 1.2 ± 0.5% (Figure 5F,J). In summary, our results reveal that the E3 site in *Barhl1* 3′-enhancer is crucial for the generation of induced hair cell-like cells in our *in vitro* induction system.

### 3.7. The E3 Box Has Similar Effects on Hair Cell Differentiation as Barhl1 Protein

**Figure 6:**
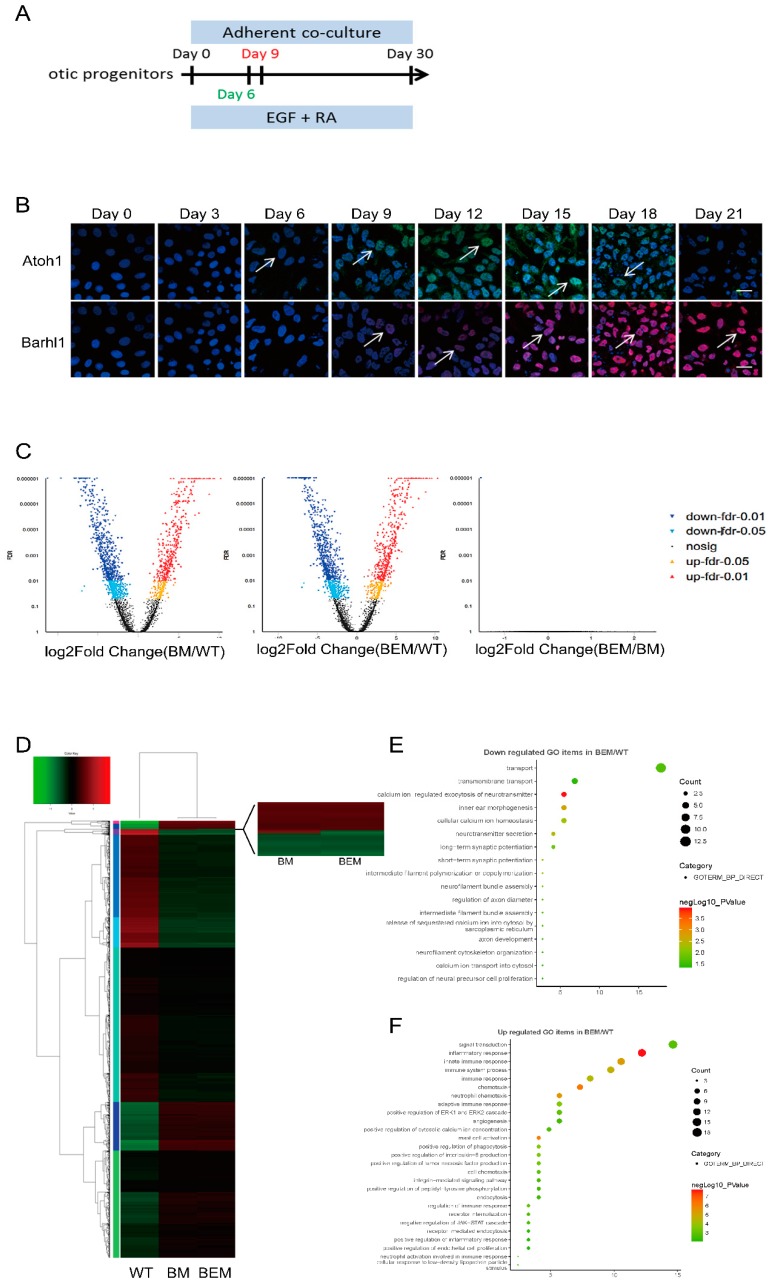
*Barhl1* is involved in hair cell differentiation and functions. (**A**) The schematic diagram shows an otic progenitors differentiation protocol with time points when *Atoh1* and *Barhl1* begins to express, marked in green and red, respectively. (**B**) Analysis of *Atoh1* and *Barhl1* expressions at different time points during in vitro induction of otic progenitors towards hair cell-like cells by immunostaining. Scale bars, 30 µm. (**C**) Differentially expressed genes-based volcano plot. Red dots and blue dots represent upregulated genes and downregulated genes in *Barhl1* CDS mutated (BM)- (left) and BEM-derived (middle) cultures relative to WT-derived cultures, respectively (|fold change| ≥ 2, *p* < 0.05). No differentially expressed genes were found in BEM derivatives relative to BM derivatives, except *Barhl1* (right). (**D**) Hierarchical clustering and heat maps display of genes expression patterns between WT-, BM-, and BEM-derived cultures; red represents above-average expression levels and green below-average levels. Each row represents a gene, and each column a sample. The right panel is an enlargement for a mutant *Barhl1* transcript detected in BM derivatives, whereas no transcripts of *Barhl1* in BEM derivatives. (**E**,**F**) Gene ontology analysis of downregulated hair cell-specific genes and upregulated inner ear non-sensory cell-specific genes in BEM derivatives relative to WT derivatives.

Barhl1 has been identified to be crucial for the hair cell differentiation both *in vivo* [25] and *in vitro* [26]. The expression of *Barhl1* has also been successfully detected in the *in vitro* system [26]. Since *Barhl1* was considered as a direct target of Atoh1 [23], to assess the validity of the *in vitro* induction system we hence investigated their expression patterns during the induction of otic progenitors towards hair cell-like cells by immunofluorescence staining (Figure 6A). And the result indicated that the onset of *Barhl1* expression occurred on day 9, and after that the level was increased and maintained during subsequent cultures (Figure 6B). Interestingly, immunostaining results also showed that the expression of *Atoh1* was upregulated around day 6, which was earlier than *Barhl1* expression, and downregulated after day 18 (Figure 6B). Both the expression patterns of *Barhl1* and *Atoh1* during *in vitro* differentiation are similar to that of *in vivo* inner auditory hair cells, which demonstrates that the *in vitro* induction system faithfully mimics the conditions *in vivo*. More importantly, the function and downstream mechanism of Barhl1 in the hair cells have been revealed *in vitro* by disrupting the coding region of *Barhl1*.

From the above data, we uncovered a critical E-box motif which mediates the transcription of *Barhl1* depending on Atoh1. The functions of Barhl1 in hair cells have been revealed *in vitro* by disrupting the coding region of *Barhl1* [26]. Thus, it is of great significance to determine how much degree dependency of *Barhl1* expression on this motif. We used the day 9 WT- and BEM-derived cells, which were induced towards hair cell-like cells from otic progenitors, to perform RNA-seq and transcriptional profiles analysis. At the same time, the RNA-seq data from BM derivatives [26] were set as a comparison. Hierarchical clustering of the transcriptome datasets of three kinds of mESC derivatives revealed a clear segregation between WT- and BM- or BEM-derived cultures (Figure 6D). As expected, BM- and BEM-derived cultures clustered in the same group indicating a close similarity in the transcriptome between these two cultures (Figure 6D). Moreover, differentially expressed genes (DEGs) were enriched in BEM and BM derivatives compared with WT derivatives. And data showed that 962 and 1006 genes were drastically downregulated in BM and BEM derivatives, respectively, while 593 and 637 genes were significantly upregulated in BM and BEM derivatives, respectively (Figure 6C). To evaluate the potential differences between mutations on the CDS region and regulatory elements, the transcriptomes from BEM and BM derivatives were also compared. No obvious DEGs were found except *Barhl1* (Figure 6C). The *Barhl1* transcripts could be detected only in BM derivatives, while not in BEM derivatives (Figure 6C,D). This was completely in conformity with their genome contexts, since BM derivatives had mutations on the coding region, which would not affect *Barhl1* expression, while BEM derivatives had mutations on the expression regulatory motif (E3-box), which mostly abolished its expression. Thus, this Atoh1 binding the *Barhl1* E3 site is very critical for driving the expression of *Barhl1* during hair cell differentiation.

To elucidate the mechanisms of hair cell differentiation in the downstream of Barhl1, we further overlapped the DEGs enriched in BEM derivatives with the RNA-seq data from developing inner ear sensory epithelium [29]. The results showed that 73 hair cell-specific genes were downregulated, while 123 inner ear non-sensory cell-specific genes were significantly upregulated in BEM derivatives (Tables S4 and S5). Gene ontology (GO) analysis via the DAVID functional annotation tool revealed that the downregulated genes were enriched in functional groups related to hair cell functions and development, such as inner ear morphogenesis, neurotransmitter secretion, synaptic potential, intermediate filament bundle assembly, intermediate filament polymerization or depolymerization, calcium ion transport, and neurofilament bundle assembly (Figure 6E), while the upregulated genes were associated with signal transduction, inflammatory response, cell chemotaxis, endocytosis, receptor internalization, and mast cell activation (Figure 6F).

## 4. Discussion

The temporal-spatial expression pattern of *Atoh1* during development [18,23,38,39] indicates its multiple developmental functions in different tissues or organs. *Atoh1* depletion leads to several types of cells loss, such as granule cells in cerebellum [40,41], goblet cells in intestine [42,43], and interneurons in spinal cord [44,45,46,47], strongly implying that *Atoh1* is an important cell lineage induction factor. *Atoh1* has been also considered as a master regulator responsible for initiating inner ear hair cell fate [11]. However, its downstream mechanisms at cellular and molecular levels are still largely unknown. Previous studies reveal that Atoh1 is an upstream activator of *Barhl1* in the cerebellum [23]. Interestingly, *Barhl1* has also been identified as a deafness gene expressed in hair cells due to its key roles in the long-term maintenance of auditory hair cells [25]. Thus, there also exists a great possibility that *Barhl1* might be a direct target of Atoh1 in auditory hair cells.

In this study, we reported the molecular mechanism underlying the regulation of *Barhl1* expression during hair cell development and revealed the molecular functions of Barhl1 in hair cells. Firstly, through comparing the 601 previously reported Atoh1 direct targetome in the cerebellum [23] and 978 DEGs in the inner ear sensory epithelium [29], we identified *Barhl1* as a direct target gene of Atoh1 in hair cells. Similar strategy has also been successfully utilized in identifying direct targets of Atoh1 in the dorsal spinal cord [39], suggesting the effectiveness of this method in identifying Atoh1 cell-specific direct targets in different tissues. Secondary, the Atoh1 ChIP-seq data identified a highly conserved Atoh1-bound region containing four E-boxes at 3’ of the *Barhl1* CDS [47]. Genomic region bound by Atoh1 does not necessarily suggest that it has enhancer activity [39]. However, previous study reported that both this conserved region and the *Barhl1* initiation codon region were bound by acetyl histone H3 during cerebellar granule cell differentiation [48], suggesting that this Atoh1-bound region had enhancer activity. Additionally, we examined the enhancer status of the E3 containing region in cerebellar cells [23] in the ENCODE project. Generally, H3K4me1 and H3K27ac are enriched in this region. H3K4me1 and H3K27ac are more enriched in the region containing four E boxes than the region without E boxes, which suggests that this region might act as an enhancer in cerebellar cells. As such, it is also possible that this region might be an enhancer in auditory hair cells as well. In this study, to further confirm this region to be an Atoh1-responsive enhancer and figure out the response of each E-box to Atoh1, we employed in vitro GFP reporter and mutational assays in HEK293T cells. Among E1-E4 sites, only the E3 site (CAGCTG) mutation inhibited the enhancer activity in the presence of *Atoh1* expression, indicating its crucial role in mediating *Barhl1* expression. Notably, the same kind of E-box (CAGCTG) motif has been reported to be bound by Atoh1 to regulate the expression of *Barhl2* [49] and *Atoh1* itself [32] in central nervous system and/or the inner ear, which indicates a significant regulatory role of E-box involving cell differentiation and development. The canonical E-box usually contains the CANNTG consensus sequence, which can be bound by different proneural bHLH proteins [50]. Atoh1 is one member of class II bHLH proteins and preferentially bind to a slightly different E-box termed AtEAM [23], which shows preference for the middle NN residues and flanking sequence. This may account for the functional differences of Atoh1 as a bHLH protein to trans-activate distinct target genes, compared with other bHLH proteins. Our results strongly suggest that Atoh1 binds to this essential AtEAM motif (CAGCTGGT) to directly activate *Barhl1* expression. Thirdly, to further investigate the regulatory mechanism controlling *Barhl1* expression in hair cells, we adopted an effective *in vitro* differentiation system [26], which provides an efficient platform for auditory hair cell-related studies. A mESC line carrying a targeted disruption introduced by Cas9 technology on the essential E-box in *Barhl1* enhancer (BEM) was established (Figure 3A–D). After testing its pluripotency, the BEM cell line was subjected to the hair cell induction program, and we found that the E3 site mutation did not affect the induction of EPL cells and subsequent differentiation of otic progenitors, most likely because of the absence of *Barhl1* expression in these induced EPL cells and otic progenitors. Studies have shown that *Barhl1* commenced its expression at least 3.5 days later than the formation of primitive ectoderm (equivalent to EPL cells) [24,51], while in the developing inner ear, *Barhl1* expression appears after formation of hair cells from otic progenitors [25]. Following the guidance towards hair cells, we found that *Barhl1* expression was absent in BEM derivatives, and the number of hair cell-like cells derived from BEM line decreased sharply relative to that of WT derivatives (Figure 5A–J). This phenomenon is similar to the loss of auditory hair cells in *Barhl1* mutant mice [25], suggesting that the E3 site mutation affects *Barhl1* expression in hair cell-like cells, thus leading to their degeneration. Combined with the GFP reporter assay and mutational analysis of E-box motif, we can conclude that *Barhl1* activation in hair cells relies on the interaction of Atoh1 with the E3 site (CAGCTG) in the *Barhl1* 3′ enhancer.

**Figure 7:**
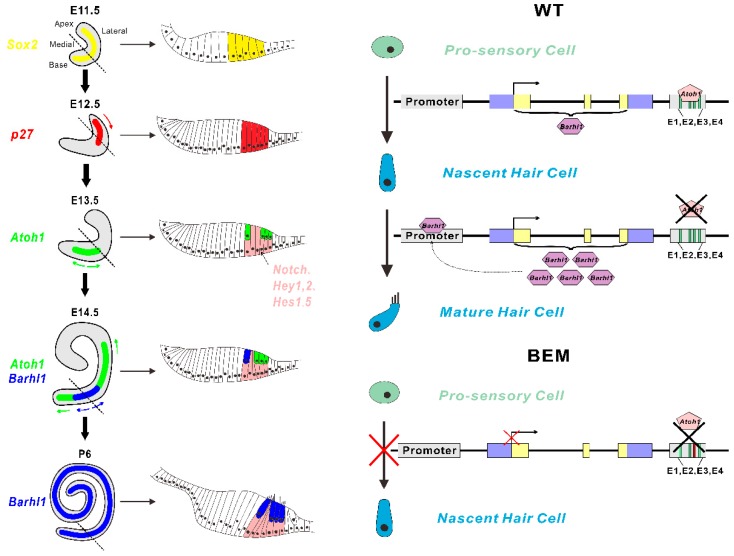
Atoh1-regulated *Barhl1* expression during the organ of Corti development. Left panel: A concise outline of the cochlear development with cross sections at corresponding periods listed next to it. The cochlear duct commences with an expansion of the ventral part of the otocyst. A diagram of the entire cochlea at E11.5 highlights the expression of *Sox2* (indicated by yellow) in the pro-sensory region. Several cyclin-dependent kinase inhibitors urge the pro-sensory cells to exit from cell cycles and this withdrawal originates from the apex and extends towards the base at E12.5. The gradient expression of *p27* (indicated by red arrow) is consistent with the direction of cell cycle exit in the cochlea. Then the pro-sensory cells are guided into hair cell fate and supporting cell fate separately by Atoh1 and Notch signaling pathways. *Atoh1* first expresses in the post mitotic pro-sensory region at E13.5, which signifies the beginning of hair cell differentiation. Then Atoh1 expands its expression from the midbase to both the apex and base of the duct bidirectionally (indicated by green arrows). Notch, Hey1,2, and Hes1,5 (indicated by pink) direct pro-sensory cells to supporting cells. For the survival and maintenance of hair cells, multiple cell signaling pathways and genes are involved. Barhl1 first appears at E14.5 in the mid-base of cochlea and its expression pattern (indicated by blue arrows) follows that of Atoh1. At P6, *Atoh1* expression ceases while *Barhl1* expression continues to maintain the normal function of hair cells. Right panel: Schematics showing Atoh1 drives *Barhl1* expression through the E3 site in the 3′ enhancer. In the WT cell line, Atoh1 binds to the E3 site located at the 3′ enhancer to activate *Barhl1* expression, thereby driving the differentiation of pro-sensory cells into nascent hair cells. When *Atoh1* stops its expression at P6, Barhl1 can bind to the promoter region to maintain its own expression to form mature hair cells. However, in the BEM cell line, since Atoh1 cannot bind to the mutated E3 site in the 3′ enhancer, the expression of *Barhl1* failed to be activated, leading to the failure of nascent hair cell differentiation from pro-sensory cells. Blue boxes, UTRs; yellow boxes, *Barhl1* coding sequence (CDS); gray boxes, promoter and enhancer; black arrow, direction of transcription.

Auditory hair cells reside in the sensory epithelium of cochlea duct. Mouse auditory hair cells differentiate from prosensory cells at around E13.5, as the cochlea duct begins to elongate (Figure 7). This process is gradual, subtle, and orchestrated by multiple molecular and cellular events [52]. After mitotic termination and initiation of *Atoh1* expression, the prosensory cells begin to separate their fates under the effects of Shh and Notch signaling, as sensory hair cells and non-sensory supporting cells. *Jag2* and *Delta1* are expressed in prospective hair cells binding on Notch1 to activate Notch pathway inside neighboring cells. Thereafter *Hes1* and *Hes5* are upregulated to repress the expression of *Atoh1*, resulting in supporting cell fate [53,54]. Instead, Shh pathway helps maintain *Atoh1* expression to trigger hair cell fate [22]. Thus, the expression of *Atoh1* in prospective hair cells is transient and begins to vanish after birth (Figure 7). It is most likely that downstream events take over the responsibility to maintain the cell fate after initiation by Atoh1. Results from us [26] and others [25,31] strongly suggest that *Barhl1* is one of such direct candidates. The stepwise auditory hair cell *in vitro* induction system precisely mimics the cell differentiation process *in vivo* and shows high efficiency for hair cell generation [26], thus providing a good opportunity to investigate molecular and cellular events during differentiation. Our result showed that the onset of *Atoh1* expression (around day 6) occurred just ahead of *Barhl1* (around day 9) and then vanished around day 18 (Figure 6B), while *Barhl1* expression was continually maintained during the subsequent cultures. This faithfully reflects the situation *in vivo* and convinces us to choose a preferable timepoint for harvesting cells to perform RNA-seq. Comparisons of transcriptomes from WT-, BM- [26], and BEM-derived cultures during hair cell induction and GO analysis revealed that 73 downregulated hair cell-specific genes in BEM derivatives were enriched in the categories associated with hair cell functions and development, such as neurotransmitter secretion, calcium ion transport, and neurofilament bundle assembly, which were consistent with a mature phenotype rather than the initial differentiation of hair cells. This again demonstrates that *Barhl1* is a downstream gene of Atoh1 and is responsible for hair cell differentiation.

Mutations on the *Barhl1* CDS exhibit severe phenotypes on hair cells both in vivo [25] and in vitro [26]. In this study, however we disrupted an E-box motif in the noncoding enhancer region of *Barhl1*, which is indispensable for Atoh1 binding. Our results revealed that the disruption of this E-box achieved the same effect on hair cell differentiation as the CDS region mutation. More significantly, this E3 box is highly conserved among mouse, rat, and human [47], implying its evolutionary and critical roles for auditory functions. Promoting mammalian auditory hair cell regeneration, like low vertebrates, is considered as one of the critical steps for auditory function restoration. Therefore, it is of great significance to explore mutation types, frequencies, and epigenetic landscapes on this site among populations with hereditary hearing impairment. Furthermore, this will also provide great benefits to the genetic and cellular therapies for deafness on genetic cues. For example, if genetic factors causing mutations and modifications at this site lead to hair cell dysfunction, Crispr/Cas9 mediated techniques can be implemented to correct the mutations, activate expression, or remove epigenetics inhibition to restore proper *Barhl1* expression. For the case of hair cell degeneration, Crispr/Cas9 mediated techniques can also be utilized to activate supporting cells to regenerate hair cells since they share the same cell lineage origin in differentiation. Certainly, cell transplantation with appropriately differentiated cells from *in vitro* induced resources will be another potential option.

## 5. Conclusions

Altogether, we demonstrate that *Barhl1* is a downstream directly target gene of Atoh1. Our findings reveal that the E3 box with an AtEAM motif (CAGCTGGT) in the 3′ enhancer of the *Barhl1* CDS is required for Atoh1-binding to mediate *Barhl1* expression. Additionally, the mutated E3 site in mESCs can result in failure of hair cell differentiation without interference with the induction of EPL cells and otic progenitors, similar to the effects of the CDS region mutation. Furthermore, the RNA-seq results and GO enrichment analysis of BEM line also demonstrate that the E3 site of *Barhl1* is indispensable during the development of auditory hair cells. This study contributes a novel genetic mechanism that Atoh1 functions as a transcriptional activator of *Barhl1* through the E3 box to achieve regulation of hair cell development, which can help us to better understand the pathogenesis of deafness and find relevant cues.

## Figures and Tables

**Table 1:** 35 selected genes of Atoh1 direct targets candidates in hair cells. List of 35 identified candidate Atoh1 direct target genes, of which 23 are significantly upregulated in hair cells and 12 are significantly downregulated in hair cells. * Genes known as deafness genes expressed in hair cells.

Candidate Genes Upregulated in Hair Cells	Candidate Genes Downregulated in Hair Cells
*Ano3* *Arfgef3* *Atoh1* Barhl1 * *Cacng2* *Chgb* *Htr3a* *Mfng* *Nceh1* *Necab3* *Nhlh1* *Pacrg* *Pcsk9* *Plch2* *Rab36* *Scg5* *Sgpp2* *Slc9a9* Srrm4 * *Tmcc2* *Ubash3b* *Wdr95* *Zbtb18*	*Adamts1* *Arhgap23* *Ccnd2* *Cntn6* *Eln* *Gatm* *Gm5089* *Lypd6* *Man1c1* *Pcca* *Ppp1r14c* *Rgs2*

## Supplementary Materials

The following are available online at https://www.mdpi.com/2073-4409/8/5/458/s1, Table S1: Predicted sgRNA off-target sites in BEM line. Table S2: The primers used in off-target examination in BEM line. Table S3: The primers used in qRT-PCR. Table S4: Downregulated hair cell-specific genes in BEM derivatives. Table S5: Upregulated inner ear non-sensory cell-specific genes in BEM derivatives. Figure S1: Off-target examination in BEM line.
