# Supplementary material for: A Critical E-box in Barhl1 3′ Enhancer Is Essential for Auditory Hair Cell Differentiation
Source: Cells. 2019 May 15;8(5):458. doi: 10.3390/cells8050458 (PMC6562609; doi:10.3390/cells8050458)
Supplement: Supplementary file 1 [file cells-08-00458-s001.zip › Supplementary Materials.docx]

**Supplementary Tables**

Table S1. Predicted sgRNA off-target sites

| Off-target site | Sequence | Score | Mismatches | Locus | Off-targeting  (Yes/No) |
| --- | --- | --- | --- | --- | --- |
| S1 | AAGCGCCGGGCACCAGCTGCAGG | 5.7 | 2MMs [1:4] | chr12: +87724876 | No |
| S2 | CAGGGCTGTGCACCAGCTGCAAG | 2.3 | 2MMs [7:9] | chr3: -105436426 | No |
| S3 | ACTGGCCAGGCACCAGCTGCAGG | 1.4 | 4MMs [1:2:13:8] | chrX: +101117924 | No |
| S4 | CCGCACCAGGCACCAGCTGCAGG | 1.4 | 4MMs [2:4:5:8] | chr17: +27699538 | No |
| S5 | CCGCACCTGGCACCAGCTGCGGG | 1.4 | 4MMs [2:4:5:8] | chr17: -43013689 | No |

Table S2. Off-target examination primers

| Off-target site | Forward primer (5'-3') | Reverse primer (5'-3') |
| --- | --- | --- |
| S1 | TGTCAAGGCAGGAAACAGCA | GCTACCCCTCTCACAAGCAG |
| S2 | ACATGCACTGACCCACTCAT | GGTGATGCTGGACCAGGAAA |
| S3 | CACAAAATCCAGGTGGCAGC | CACAGCCAGGAAGCACAAAC |
| S4 | GGCTGAGAATGAAGGCCAGT | CTGGGCCTTGTCAGAGTGAG |
| S5 | GGACCCCGCCTTGAACTT | AGCTGGCTGAGGCACTTG |

Table S3. Primers used in gene expression analysis

| Gene | Forward primer (5'-3') | Reverse primer (5'-3') |
| --- | --- | --- |
| *Gapdh* | TGC GAC TTC AAC AGC AAC TC | ATG AGG TCC ACC ACC CTG T |
| *Barhl1* | GAA CCG CAG GAC TAA ATG GA | AGA AAT AAG GCG ACG GGA AC |
| *Sox2* | TTT GTC CGA GAC CGA GAA GC | ATC ATG CTG TAG CTG CCG TT |
| *Nanog* | TAC CTC AGC CTC CAG CAG AT | CAC TGG TTT TTC TGC CAC CG |
| *Nestin* | GGT AGG GCT AGA GGA CCC AA | AGC CCT TGC ATT CCA GAG TC |
| *Oct4* | TGG ATC CTC GAA CCT GGC TA | CTC AGG CTG CAA AGT CTC CA |
| *Nkx2.5* | CAC ATT TTA CCC GGG AGC CT | ACC AGA TCT TGA CCT GCG TG |
| *Gbx2* | GCA ACT TCG ACA AAG CCG AG | TGA CTC GTC TTT CCC TTG CC |
| *NF-L* | AGT TTC ACC AGC GTG GGT AG | GGT CTC CTC GAC CTC TGT CT |
| *ζ-globin* | ACC ATG GGT CTC AGC AGT TG | GGA GCT TGA AGT TGA CCG GA |
| *TTR* | GGC TTC CCT TCG ACT CTT CC | CGG ACA GCA TCC AGG ACT TT |
| *AFP* | TGG TTA CAC GAG GAA AGC CC | TCC TCA GAG AAT GGG GGA GG |
| *Fgf5* | GTA CGT GGC CCT GAA CAA GA | CTG TAC TTC ACT GGG CTG GG |
| *Brn3c* | AGG GCG ACT TAC TTG AGC AC | CTC TCG AGG GTC TGA CTC CA |
| *Brachyury* | GGC TGG GAG CTC AGT TCT TT | TGT CCA CGA GGC TAT GAG GA |
| *Dlx5* | GGC CGC TTT ACA GAG AAG GT | GGT GAC TGT GGC GAG TTA CA |
| *Pax2* | TAC TCT CTC CCA GCC CTG AC | GTA CGT CTG TGT GCC TGA CA |
| *Pax8* | AAC AGT GCC CTG GAT GAT GG | GAG GAA GGG GTG GAG CTA GA |
| *Six1* | CAC GCC AGG AGC TCA AAC TA | ACC CAA GTC CAC CAA ACT GG |
| *Eya1* | CCC AGC ATT GGC AAA AGT CC | GCA TGA TGC AGT GCC ATG AG |
| *Espin* | GGT CTC AGC CAC TGC TCA ATG | GAA TGT CTC GTC TCC AGG CAG |
| *Myo7a* | AAT CAC ATC AGG TAC AGC GAA GA | CGG GGA AGT AGA CCT TGT GGA |
| *Chrna9* | GCA GTG CAA CCT GAC CTT TG | CAT GCC CTG AAC CTC CCA TT |

Table S4. Downregulated hair cell-specific genes in BEM derivatives

| Gene | Seq_id | Gene | Seq_id |
| --- | --- | --- | --- |
| *Car8* | ENSMUSG00000041261 | *Krt20* | ENSMUSG00000035775 |
| *Syt6* | ENSMUSG00000027849 | *Grtp1* | ENSMUSG00000038515 |
| *Snap25* | ENSMUSG00000027273 | *Kcnh7* | ENSMUSG00000059742 |
| *Megf11* | ENSMUSG00000036466 | *Abcc8* | ENSMUSG00000040136 |
| *Tcerg1l* | ENSMUSG00000091002 | *Rimklb* | ENSMUSG00000040649 |
| *Slc8a2* | ENSMUSG00000030376 | *A530016L24Rik* | ENSMUSG00000043122 |
| *Ptprn2* | ENSMUSG00000056553 | *Mapk15* | ENSMUSG00000063704 |
| *1810041L15Rik* | ENSMUSG00000062760 | *Hook1* | ENSMUSG00000028572 |
| *Dscaml1* | ENSMUSG00000032087 | *Actr3b* | ENSMUSG00000056367 |
| *Nefm* | ENSMUSG00000022054 | *Unc79* | ENSMUSG00000021198 |
| *Rasd2* | ENSMUSG00000034472 | *Clip4* | ENSMUSG00000024059 |
| *Rims2* | ENSMUSG00000037386 | *Tspan13* | ENSMUSG00000020577 |
| *Fcho1* | ENSMUSG00000070000 | *Gpr4* | ENSMUSG00000044317 |
| *Acsl6* | ENSMUSG00000020333 | *Ush1g* | ENSMUSG00000045288 |
| *Dmkn* | ENSMUSG00000060962 | *Ccdc33* | ENSMUSG00000037716 |
| *Syt7* | ENSMUSG00000024743 | *Rab3b* | ENSMUSG00000003411 |
| *Rassf10* | ENSMUSG00000098132 | *Rab15* | ENSMUSG00000021062 |
| *Slc4a8* | ENSMUSG00000023032 | *Syt14* | ENSMUSG00000016200 |
| *Chgb* | ENSMUSG00000027350 | *Ccdc108* | ENSMUSG00000047021 |
| *Btn2a2* | ENSMUSG00000053216 | *Foxj1* | ENSMUSG00000034227 |
| *Ablim2* | ENSMUSG00000029095 | *Dyrk3* | ENSMUSG00000016526 |
| *Stac2* | ENSMUSG00000017400 | *Trdn* | ENSMUSG00000019787 |
| *Nkx3-1* | ENSMUSG00000022061 | *Tmprss6* | ENSMUSG00000016942 |
| *Fgf21* | ENSMUSG00000030827 | *Abcd2* | ENSMUSG00000055782 |
| *Jag2* | ENSMUSG00000002799 | *Tmtc4* | ENSMUSG00000041594 |
| *Nup210* | ENSMUSG00000030091 | *Fbxo2* | ENSMUSG00000041556 |
| *Mlc1* | ENSMUSG00000035805 | *Calca* | ENSMUSG00000030669 |
| *Nefl* | ENSMUSG00000022055 | *Fhit* | ENSMUSG00000060579 |
| *Kcnb1* | ENSMUSG00000050556 | *Gbx2* | ENSMUSG00000034486 |
| *Tmem179* | ENSMUSG00000054013 | *Disp2* | ENSMUSG00000040035 |
| *Ryr2* | ENSMUSG00000021313 | *Tcp11* | ENSMUSG00000062859 |
| *Nos1ap* | ENSMUSG00000038473 | *Zcchc18* | ENSMUSG00000031428 |
| *Scg5* | ENSMUSG00000023236 | *Card10* | ENSMUSG00000033170 |
| *Gfi1* | ENSMUSG00000029275 | *Tmie* | ENSMUSG00000049555 |
| *Trim71* | ENSMUSG00000079259 | *Raver2* | ENSMUSG00000035275 |
| *Camk2b* | ENSMUSG00000057897 | *Fry* | ENSMUSG00000056602 |
| *Sall1* | ENSMUSG00000031665 |  |  |

Table S5. Upregulated inner ear nonsensory cell-specific genes in BEM derivatives

| Gene | Seq_id | Gene | Seq_id |
| --- | --- | --- | --- |
| *C1qa* | ENSMUSG00000036887 | *Myl9* | ENSMUSG00000067261 |
| *Ctss* | ENSMUSG00000038642 | *Col8a1* | ENSMUSG00000020077 |
| *Actg2* | ENSMUSG00000059430 | *Col5a2* | ENSMUSG00000022665 |
| *C1qc* | ENSMUSG00000036896 | *Entpd1* | ENSMUSG00000064080 |
| *Lyz2* | ENSMUSG00000069516 | *Ptgfr* | ENSMUSG00000059089 |
| *Ms4a7* | ENSMUSG00000024672 | *Aspn* | ENSMUSG00000037060 |
| *Tyrobp* | ENSMUSG00000030579 | *Ccl6* | ENSMUSG00000021457 |
| *Csf1r* | ENSMUSG00000024621 | *Aard* | ENSMUSG00000002020 |
| *Fcrls* | ENSMUSG00000015852 | *Cav2* | ENSMUSG00000031613 |
| *C3ar1* | ENSMUSG00000040552 | *Fcgr3* | ENSMUSG00000038151 |
| *Ptprc* | ENSMUSG00000026395 | *Crip1* | ENSMUSG00000030921 |
| *Ms4a6d* | ENSMUSG00000024679 | *Cspg4* | ENSMUSG00000021319 |
| *C5ar1* | ENSMUSG00000049130 | *Gjb2* | ENSMUSG00000018930 |
| *Tfpi2* | ENSMUSG00000029664 | *Cd52* | ENSMUSG00000033377 |
| *Dock2* | ENSMUSG00000020143 | *S100a4* | ENSMUSG00000030148 |
| *Itgbl1* | ENSMUSG00000032925 | *Nrn1* | ENSMUSG00000031385 |
| *Cav1* | ENSMUSG00000007655 | *Itga11* | ENSMUSG00000020053 |
| *Tlr7* | ENSMUSG00000044583 | *Cd68* | ENSMUSG00000032766 |
| *Lilrb4a* | ENSMUSG00000062593 | *Ly86* | ENSMUSG00000022220 |
| *Pik3cg* | ENSMUSG00000020573 | *Sla* | ENSMUSG00000060063 |
| *Mrc1* | ENSMUSG00000026712 | *Slc2a10* | ENSMUSG00000043832 |
| *Il10ra* | ENSMUSG00000032089 | *Slc25a45* | ENSMUSG00000056481 |
| *Tnmd* | ENSMUSG00000031250 | *Gpx7* | ENSMUSG00000039476 |
| *Tnfrsf11a* | ENSMUSG00000026321 | *Rinl* | ENSMUSG00000031712 |
| *Fcer1g* | ENSMUSG00000058715 | *Cxcl16* | ENSMUSG00000024168 |
| *Lcp2* | ENSMUSG00000002699 | *Foxd3* | ENSMUSG00000067261 |
| *Cyth4* | ENSMUSG00000018008 | *Srgn* | ENSMUSG00000020077 |
| *Gatm* | ENSMUSG00000027199 | *Ccdc80* | ENSMUSG00000022665 |
| *Cd53* | ENSMUSG00000040747 | *Fbln2* | ENSMUSG00000064080 |
| *Pcolce2* | ENSMUSG00000015354 | *Fcgr4* | ENSMUSG00000059089 |
| *Ctse* | ENSMUSG00000004552 | *Prkcdbp* | ENSMUSG00000037060 |
| *Slc5a7* | ENSMUSG00000023945 | *Syk* | ENSMUSG00000021457 |
| *Fbln5* | ENSMUSG00000021186 | *Ltbp2* | ENSMUSG00000002020 |
| *Msr1* | ENSMUSG00000025044 | *Hpgd* | ENSMUSG00000031613 |
| *Tagln* | ENSMUSG00000032085 | *Prdm1* | ENSMUSG00000038151 |
| *Fyb* | ENSMUSG00000022148 | *Trim30a* | ENSMUSG00000030921 |
| *Lgals3* | ENSMUSG00000050335 | *Sfrp4* | ENSMUSG00000021319 |
| *Stab1* | ENSMUSG00000042286 | *Ccl4* | ENSMUSG00000018930 |
| *Lair1* | ENSMUSG00000055541 | *Palmd* | ENSMUSG00000033377 |
| *Cd48* | ENSMUSG00000015355 | *Clec4a2* | ENSMUSG00000030148 |
| *Mpeg1* | ENSMUSG00000046805 | *Plxnb3* | ENSMUSG00000031385 |
| *Plek* | ENSMUSG00000020120 | *Igf1* | ENSMUSG00000020053 |
| *Ccr5* | ENSMUSG00000079227 | *Gng11* | ENSMUSG00000032766 |
| *Pon3* | ENSMUSG00000029759 | *Adcy4* | ENSMUSG00000022220 |
| *Lama2* | ENSMUSG00000019899 | *Alox5ap* | ENSMUSG00000060063 |
| *Npy* | ENSMUSG00000029819 | *Clec4a3* | ENSMUSG00000043832 |
| *Arhgap30* | ENSMUSG00000048865 | *Cd248* | ENSMUSG00000056481 |
| *P2ry12* | ENSMUSG00000036353 | *Prrx2* | ENSMUSG00000039476 |
| *Cybb* | ENSMUSG00000015340 | *Il15* | ENSMUSG00000031712 |
| *Srpx* | ENSMUSG00000090084 | *Tmem204* | ENSMUSG00000024168 |
| *Adamts5* | ENSMUSG00000022894 | *Gdf10* | ENSMUSG00000021943 |
| *Lcp1* | ENSMUSG00000021998 | *Lrrc4c* | ENSMUSG00000050587 |
| *Ly6a* | ENSMUSG00000075602 | *Cd36* | ENSMUSG00000002944 |
| *Sox10* | ENSMUSG00000033006 | *Slit2* | ENSMUSG00000031558 |
| *Hmha1* | ENSMUSG00000035697 | *Stat6* | ENSMUSG00000002147 |
| *Gpr65* | ENSMUSG00000021886 | *Nmi* | ENSMUSG00000026946 |
| *Nov* | ENSMUSG00000037362 | *Timp3* | ENSMUSG00000020044 |
| *Gpr183* | ENSMUSG00000051212 | *Col24a1* | ENSMUSG00000028197 |
| *Cdh19* | ENSMUSG00000047216 | *Cd200* | ENSMUSG00000022661 |
| *Thbd* | ENSMUSG00000074743 | *Crispld1* | ENSMUSG00000025776 |
| *Bgn* | ENSMUSG00000031375 | *Cyba* | ENSMUSG00000006519 |
| *Ms4a6c* | ENSMUSG00000079419 |  |  |

**Supplementary Figure S1**

**
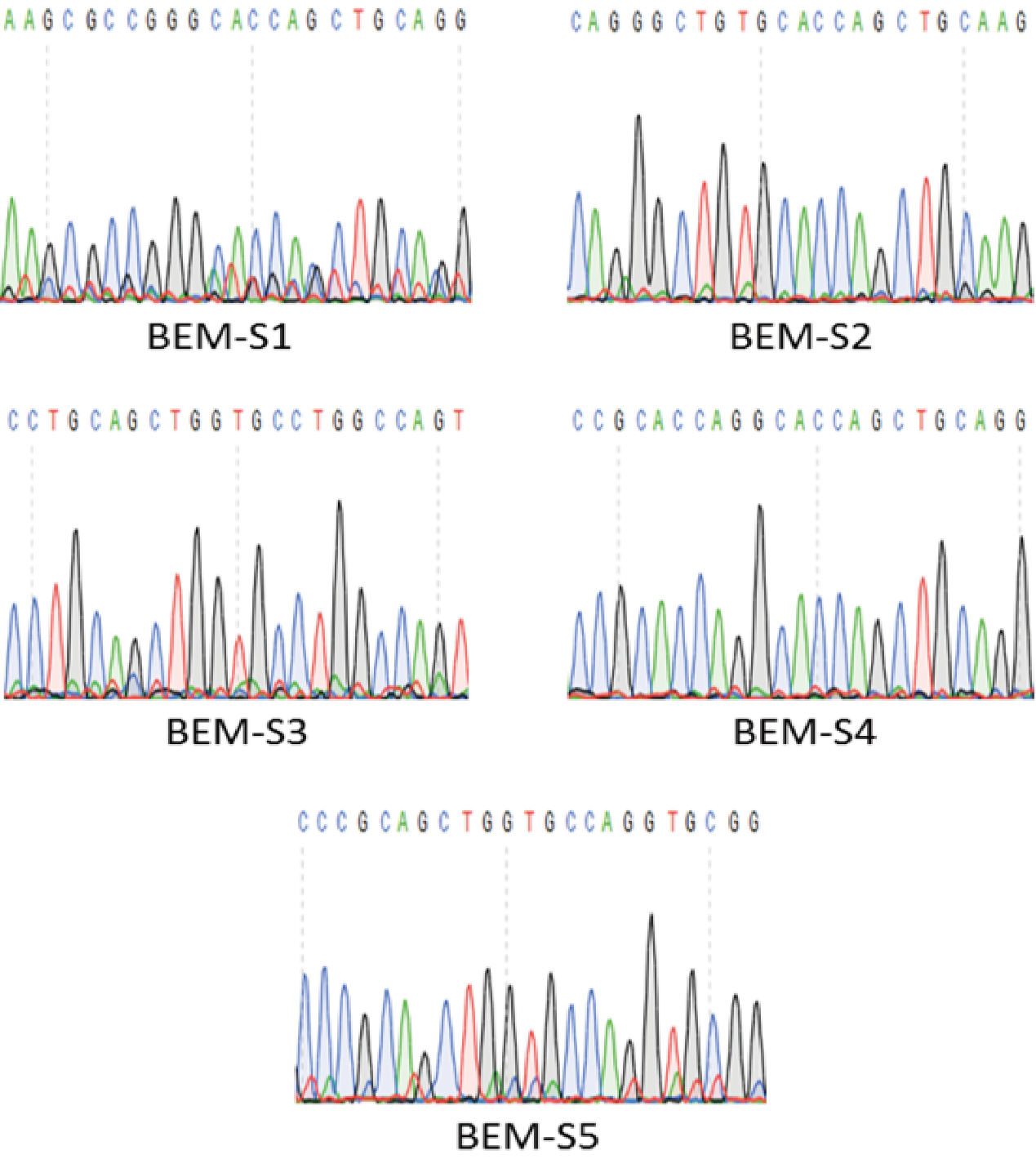
**

**Figure S1.** **Off-target examination of** **BEM mESC line.** The BEM line was sent for Sanger sequencing to examine potential off-targeting. No off-targeting was shown by no double peaks adjacent to the top 5 most likely off-target sites.
